# A Fatal Case of Coronavirus Disease 2019 (COVID-19) in a Patient With Idiopathic Pulmonary Fibrosis

**DOI:** 10.7759/cureus.8432

**Published:** 2020-06-03

**Authors:** Venkat Rajasurya, Kulothungan Gunasekaran, Vijay Damarla, Anuradha Kolluru

**Affiliations:** 1 Pulmonary Critical Care, Novant Health, Winston-Salem, USA; 2 Pulmonary Critical Care, Yale New Haven Health at Bridgeport Hospital, Bridgeport, USA; 3 Hematology and Oncology, Decatur Memorial Hospital, Decatur, USA; 4 Cardiology, Decatur Memorial Hospital, Decatur, USA

**Keywords:** covid-19, corona virus, ipf, idiopathic pulmonary fibrosis, acute hypoxemic respiratory failure

## Abstract

The number of cases of coronavirus disease 2019 (COVID-19) has been exponentially increasing everyday. It is important to recognize the comorbidities and risk factors associated with this highly contagious and serious disease that has caused thousands of deaths worldwide. Patients with certain conditions like diabetes, hypertension, cardiovascular disease and chronic lung diseases have been reported to develop serious complications from COVID-19. Idiopathic pulmonary fibrosis (IPF) is a disease that is more prevalent in the elderly population, the same group that are more susceptible to serious complications from COVID-19. Our literature search did not reveal any review about COVID-19 in IPF patients. We report a patient with IPF who was exposed to COVID-19 from her spouse and died from its complications. This case would help to raise the awareness among IPF patients to follow the necessary precautions to reduce the risk of contracting the disease.

## Introduction

Coronavirus disease 2019 (COVID-19) is a rapidly spreading infectious disease caused by novel severe acute respiratory syndrome coronavirus-2 (SARS-CoV-2) [1]. This pandemic has already led to thousands of deaths worldwide, and the number of infected cases continues to rise everyday. Although this virus could infect anyone, it has been found that patients who are older and those with certain pre-existing comorbidities suffer from serious complications due to this disease. Idiopathic pulmonary fibrosis (IPF) is a chronic progressive interstitial lung disease of unknown etiology and its prevalence is higher in the elderly population. Chronic lung disease has been recognized as a risk factor for serious COVID-19 disease, and we report a case of fatal COVID-19 viral pneumonia in a patient with IPF [1-3]. 

## Case presentation

**Figure 1:**
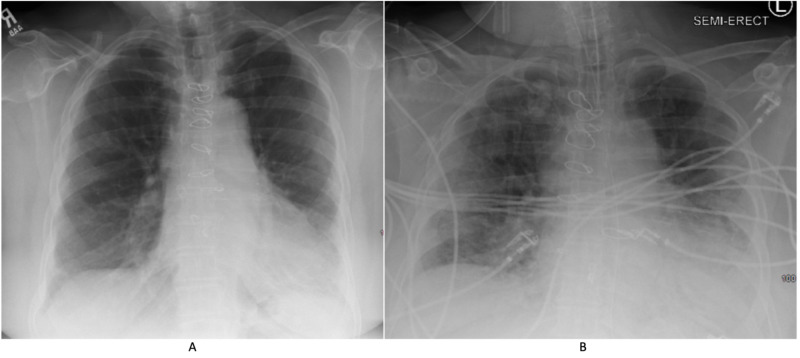
Chest X-ray (A) Chest X-ray done three months prior to presentation shows bilateral interstitial infiltrates predominantly in the lung bases. (B) Chest X-ray done on day 4 of COVID-19 shows worsening bilateral multifocal infiltrates superimposed on chronic changes.

On April 2, 2020, a 79-year-old female with a history of hypertension and IPF presented with cough, worsening dyspnea, increased oxygen requirements, fever and diarrhea. Her vital signs include a temperature of 100.4°F, a heart rate of 98 bpm, an oxygen saturation of 85% on room air and a blood pressure of 102/56 mmHg. She is usually on oxygen two liters per minute at home for chronic hypoxic respiratory failure from IPF. She was on nintedanib 100 mg twice daily for her IPF and metoprolol 12.5 mg daily for her hypertension. Her pulmonary function test done two years ago showed moderate restrictive lung disease with a forced vital capacity of two liters (77% predicted), total lung capacity of three liters (63% predicted) and diffusion capacity of 58% predicted. Her physical exam was unremarkable. She was placed on isolation precautions because of her clinical presentation and her test for SARS-CoV-2 came back positive (Roche’s Cobas nucleic acid amplification test). She was exposed to her husband who was recently diagnosed with COVID-19. She required three liters of supplemental oxygen for her hypoxemia. Chest radiology showed multifocal consolidations in both lungs (Figure 1).

She received azithromycin and hydroxychloroquine as per the protocol. However, her condition deteriorated requiring 10 liters of supplemental oxygen and on day 3 she was transferred to intensive care unit and intubated for worsening hypoxic respiratory failure. She required 100% fractional concentration of oxygen in inspired gas (FiO_2_) and 10 cm H_2_O positive end-expiratory pressure (PEEP) on the ventilator. A limited bedside echocardiogram revealed normal left and right ventricular function. Her inflammatory markers continued to worsen. Ferritin which was 600 ng/mL at the time of her presentation, worsened to 50,480 ng/mL. Her white blood cell (WBC) count was 15,000/mm^3 ^with an absolute lymphocyte count of 800/mm^3^, procalcitonin was 0.14 ng/mL, D-dimer was 3.3 µg/mL and lactate dehydrogenase (LDH) was 628 IU/L. She received a single intravenous dose of tocilizumab 600 mg. On day 4, she developed severe septic shock and multiorgan dysfunction (worsening renal function, liver function, circulatory collapse and respiratory failure) requiring norepinephrine, vasopressin, phenylephrine and angiotensin II. She was started on intravenous hydrocortisone 100 mg three times daily, but despite all the aggressive measures she died on day 5. We would like to mention that at the time of this patient's presentation, other treatments like convalescent plasma and remdesivir were not readily available. 

## Discussion

Chronic lung disease has been reported as a potential risk factor for COVID-19 caused by SARS-CoV-2. In a study that looked at the clinical characteristics and comorbidities in more than 44,000 patients infected with SARS-CoV-2 in China, chronic lung disease had been listed as a comorbidity in only 2.4% of them [1]. On April 8 2020, Centers for Disease Control (CDC) released an early report on the characteristics of hospitalized patients with COVID-19 in the United States, and chronic lung disease was reported as a risk factor in 34.6% of the 159 hospitalized COVID-19 patients [2]. It was also reported that 80% of these patients had obstructive lung disease. On April 29 2020, in the data released by CDC, out of 305 COVID-19 patients in Georgia, chronic respiratory disease was reported as a comorbidity in 20.3% and out of whom 77% had obstructive airway diseases [3]. The prevalence of COVID-19 in IPF has not been reported. IPF is the most common type of idiopathic interstitial pneumonia, characterized by progressive fibrosis with a high fatality rate. The incidence and prevalence of the disease increase with age. IPF is characterized by inflammation and lung injury, and the role of cytokines in its pathogenesis has been well established [4]. There is accumulating evidence that a subgroup of patients with COVID-19 develop cytokine storm syndrome leading to increased mortality from the virus-induced hyperinflammation [5]. It is possible that co-existence of these two fatal disease conditions may significantly affect patient outcomes. On the other hand, survivors of COVID 19 have been reported to develop pulmonary fibrosis as a consequence of dysregulated immune response [6].

## Conclusions

Patients living with IPF may not be at increased risk for contracting COVID-19, but probably are at high risk for developing severe fatal complications from the disease. Hence, it is vital to recognize IPF as a potential serious risk factor for COVID-19 complications and IPF patients take the necessary extra precautions advised by CDC to protect themselves and minimize the risk of contracting COVID-19.
